# Beyond Binary: Gender Reassignment in a Case of 11β-Hydroxylase Deficiency

**DOI:** 10.7759/cureus.48644

**Published:** 2023-11-11

**Authors:** Mohammed Afsharhussain Hithayathulla, Hrithik Dakssesh Putta Nagarajan, Vrijesh Gopalakrishnan, Kaargil Puliyadi Rishi, Gopalakrishnan Chandrasekaran

**Affiliations:** 1 Department of Internal Medicine, Madurai Medical College, Madurai, IND; 2 Department of Urology, Nithilaa Nursing Home, Madurai, IND

**Keywords:** hypertension, adrenal glands, gender reassignment surgery, hematuria, congenital adrenal hyperplasia, case report

## Abstract

Congenital adrenal hyperplasia (CAH) encompasses a spectrum of disorders characterized by enzyme deficiencies in the hormone biosynthesis pathways of the adrenal glands, resulting in impaired cortisol synthesis. These disorders are typically inherited in an autosomal recessive pattern. Numerous enzymes participate in the hormonal synthesis within the adrenal glands, and the clinical presentation of affected individuals exhibits significant variability, contingent upon the specific enzyme deficiency and its severity. In this case, we present a compelling instance of 11β-hydroxylase deficiency (11βOHD). The patient initially presented as a male, with complaints of early-onset hypertension and intermittent hematuria. He had a history of precocious puberty and had experienced a progressive increase in breast size. Subsequently, the patient was found to have an XX karyotype, and a pelvic ultrasound revealed the presence of a uterus, two ovaries, and a rudimentary vagina. Gender reassignment surgery was done to this patient. This intricate case underscores the critical importance of promptly recognizing and effectively managing CAH. Timely and appropriate treatment is pivotal in ensuring the well-being of affected individuals.

## Introduction

Congenital adrenal hyperplasia (CAH) groups together the rare genetic disorders that affect the hormonogenesis of the adrenal glands. 21-Hydroxylase deficiency (21OHD) is the most common variant of CAH accounting for about 90-99% of CAH cases. This is followed by 11β-hydroxylase deficiency (11βOHD), which constitutes 0.2-8% of CAH cases. The pathogenesis of 11βOHD involves decreased functionality or deficiency of the 11β-hydroxylase enzyme. This enzyme plays a pivotal role in the conversion of 11-deoxycortisol to cortisol and 11-deoxycorticosterone to corticosterone within the adrenal cortex. Consequently, 11βOHD leads to an accumulation of 11-deoxycortisol and 11-deoxycorticosterone, with the surplus precursors being redirected towards androgen production. This results in a relative excess of androgens [1].

These hormonal alterations manifest in several ways, including virilization of female neonates and precocious puberty due to the excess androgens (testosterone and androstenedione). Early-onset hypertension is another common feature due to the excess of 11-deoxycorticosterone, which possesses a mild mineralocorticoid action, and is documented as a very rare cause of mineralocorticoid-induced hypertension. In rare cases, electrolyte abnormalities such as hypokalemia may also occur [1,2]. Herein, we present a case of 11βOHD, with all these features. Additionally, we provide insights into the intermittent hematuria experienced by this patient, shedding light on this particular aspect of the condition.

## Case presentation

**Figure 1:**
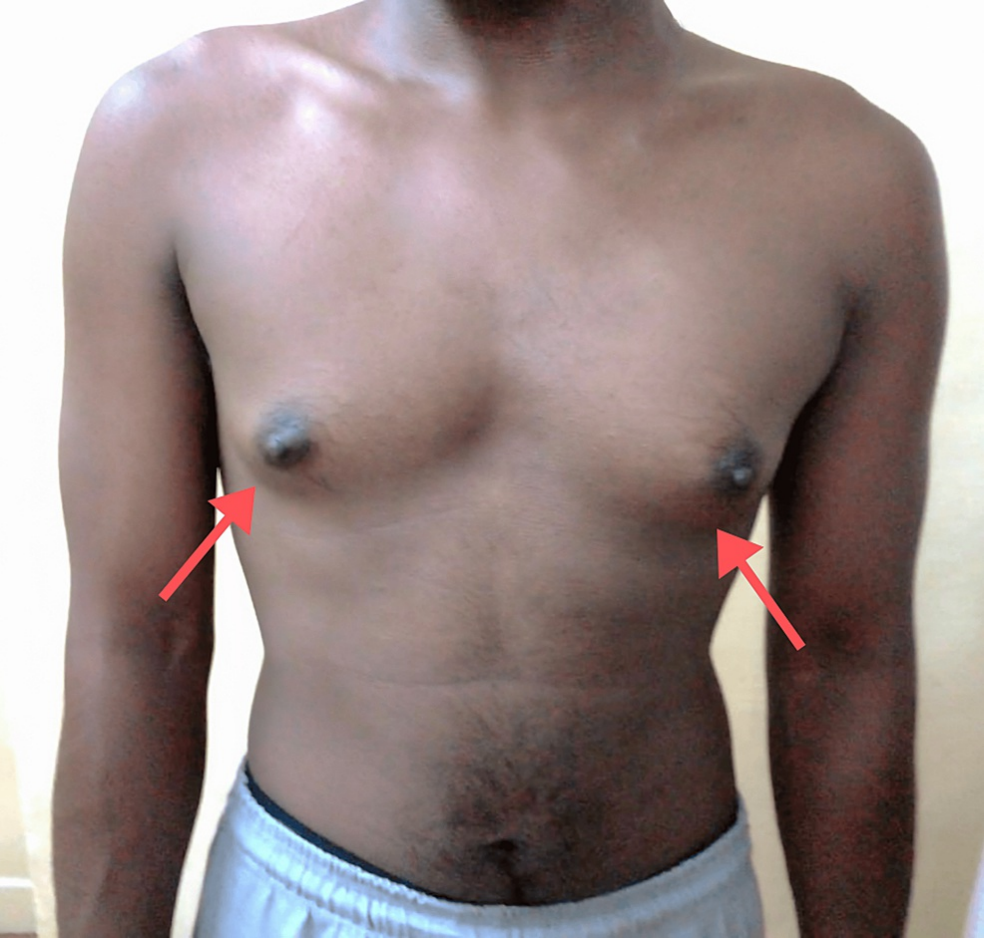
Clinical image showing the enlargement of the breasts (Tanner stage 3).

A 20-year-old South Asian patient, identifying as a male, presented to the clinic with complaints of intermittent reddish tinge to the urine over the past six months. He described that such change in the color of urine appeared to be cyclical in nature, occurring approximately once a month, with each episode lasting for about three to five days. He also expressed concerns about progressive breast enlargement (Figure 1). This patient was incidentally detected to have hypertension two years ago and was started on oral antihypertensive therapy with nifedipine (60 mg/day). 

On examination of the patient, generalized hyperpigmentation and a short stature were noted. Also, presence of facial hair, axillary hair, and pubic hair (Tanner stage 5) was noted. Furthermore, significant breast enlargement (Tanner stage 3) was also noted. Examination of the genitalia revealed normal pigmentation and rugosity. The phallus measured 10 cm with a single opening at its tip. Bilateral labioscrotal folds were noted to be fused. Bilateral testes were not palpable either in the labioscrotal folds or in the abdomen. Blood pressure was found to be 140/80 mmHg in the right upper limb in a supine posture. Routine blood investigations revealed decreased levels of potassium.

Calculations based on the heights of the patient's parents yielded an expected midparental height of 152 cm for a male offspring and 139 cm for a female offspring. However, the patient's measured height was only 140 cm, falling below the third percentile when compared to the midparental height calculation. 

On further inquiry, the patient was found to not have any developmental delays. Instead, a history of earlier development of pubic, axillary, and facial hair and increase in the size of the penis was unveiled, pointing towards precocious puberty. These changes started to occur in this patient as early as seven to nine years of age. Upon exploring the patient's family history, it was discovered that he was born out of a third-degree consanguineous marriage. No gonads were palpable at the time of his birth. However, this complaint was not brought under medical attention. Also, the elder brother of this patient had young-onset hypertension, short stature, and precocious puberty. 

Investigations

**Figure 2:**
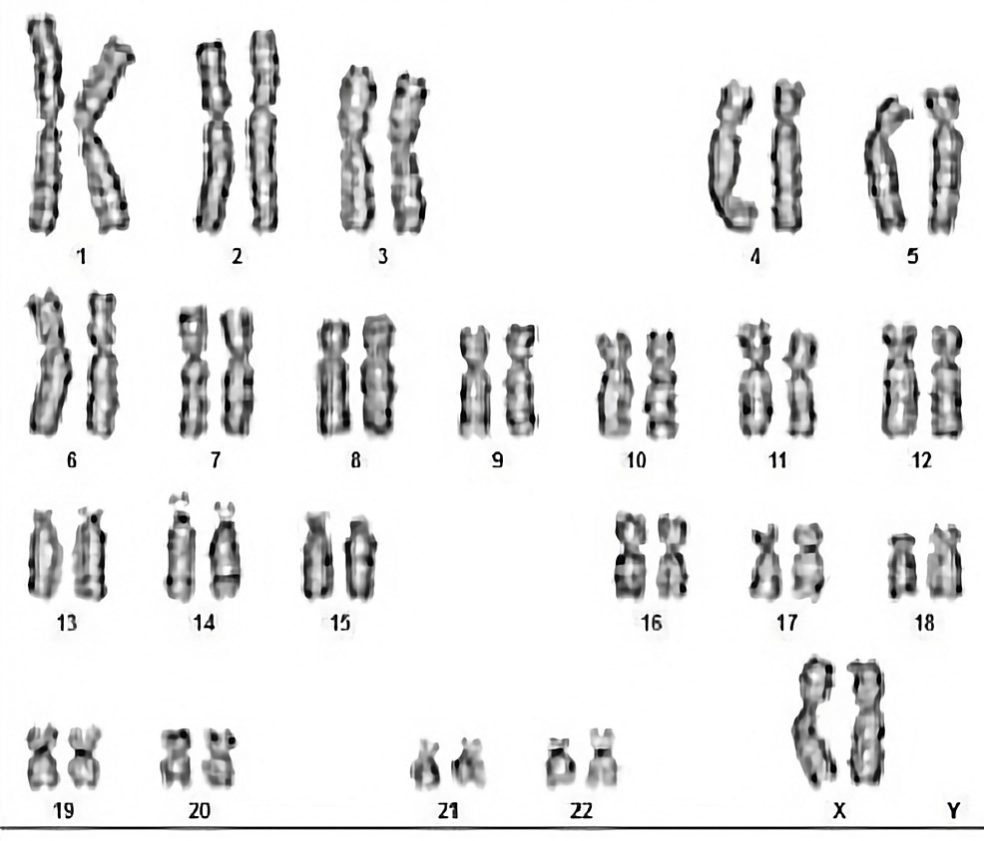
Report of the karyotype analysis. Analysis revealed 44 autosomes and the presence of two X chromosomes (female genotype).

All these clues clinched towards a possibility of CAH. So, an extensive blood workup, including the serum levels of adrenal hormones and their precursors, was ordered (Table 1). The adrenal hormonal profile was estimated using liquid chromatography and mass spectrometry, which is considered as one of the best methods of steroid profile estimation. Elevated levels of adrenal hormone precursors, namely, 11-deoxycortisol and 11-deoxycorticosterone, and 17-hydroxyprogesterone was noted. Karyotyping was done by GTG banding with trypsin and Giemsa with 450-550 band patterns (ISCN-2016). Analysis revealed 44 autosomes and the presence of two X chromosomes (female genotype) (Figure 2). Unfortunately, genetic analysis could not be done due to financial constraints. Still, the cyclical hematuria remained largely unexplained necessitating further imaging studies of the abdomen and pelvis. 

**Table 1:** Extensive blood workup findings including serum levels of adrenal hormones and their precursors. The serum levels of cortisone and corticosterone are decreased. On the other hand, the serum levels of 11-deoxycortisol, 11-deoxycorticosterone, and 17-hydroxyprogesterone are increased.

Parameter	Value	Reference values
Hemoglobin (g/dL)	14.6	12-15
Serum urea (mg/dL)	19	20-40
Serum creatinine (mg/dL)	0.8	0.3-1.2
Serum sodium (meq/L)	138	135-145
Serum potassium (meq/L)	2.6	3.5-5.5
Serum free T4 (ng/dL)	1.5	0.9-1.7
Serum thyroid-stimulating hormone (μIU/mL)	3.25	0.27-4.2
Serum luteinizing hormone (mIU/mL)	0.8	Male: 1.7-8.5; female: 2.4-12.6
Serum follicle-stimulating hormone (mIU/mL)	2.75	Male: 1.5-12.4; female: 3.5-12.5
Serum testosterone (ng/dL)	589.5	Male: 249-838; female: 2.9-40
Serum estradiol (pg/mL)	57.29	Male: 25-80; female: 12.4-233
Serum cortisol 8 am (μg/L)	6.54	5-25
Serum cortisone (μg/L)	1.57	6-27
Serum corticosterone (μg/L)	0.15	1-20
Serum 11-deoxycortisol (μg/L)	248	0.5-3
Serum 11-deoxycorticosterone (μg/L)	8.6	0.02-0.15
Serum 17-hydroxyprogesterone (μg/L)	5.18	0.2-2.2
Serum dehydroepiandrosterone (μg/dL)	2670	99-5154

Ultrasonography

**Figure 3:**
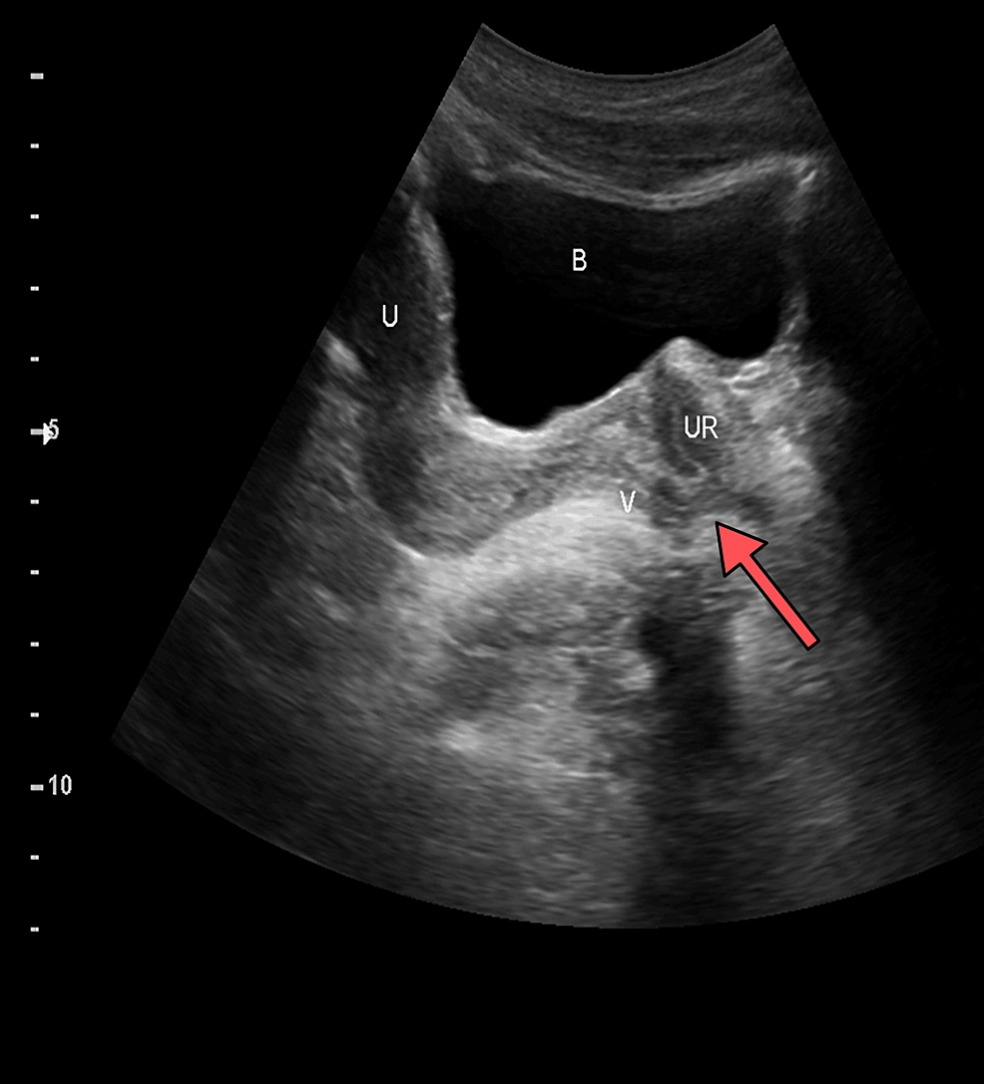
Ultrasound imaging of the pelvis revealing various structures. The abnormal connection between the lower part of the vagina and the posterior part of the urethra can be seen (red arrow). B: urinary bladder; U: uterus; V: vagina; UR: ureter

Ultrasonography revealed bilateral enlarged, hyperplastic adrenal glands. Normal Mullerian structures including the uterus and two ovaries with follicles were clearly demonstrated. Also, the upper part of the vagina was clearly visualized. The uterus measured 5 x 2.7 x 2.2 cm, and both the ovaries measured 2.3 x 1.1 cm. The lower part of the vagina appeared to communicate with the posterior part of the urethra (Figure 3). The scrotum was empty, and no testes could be identified neither in the scrotum nor in the inguinal region. Additionally, bilateral medullary nephrocalcinosis was also noted. 

Magnetic Resonance Imaging (MRI) 

**Figure 4:**
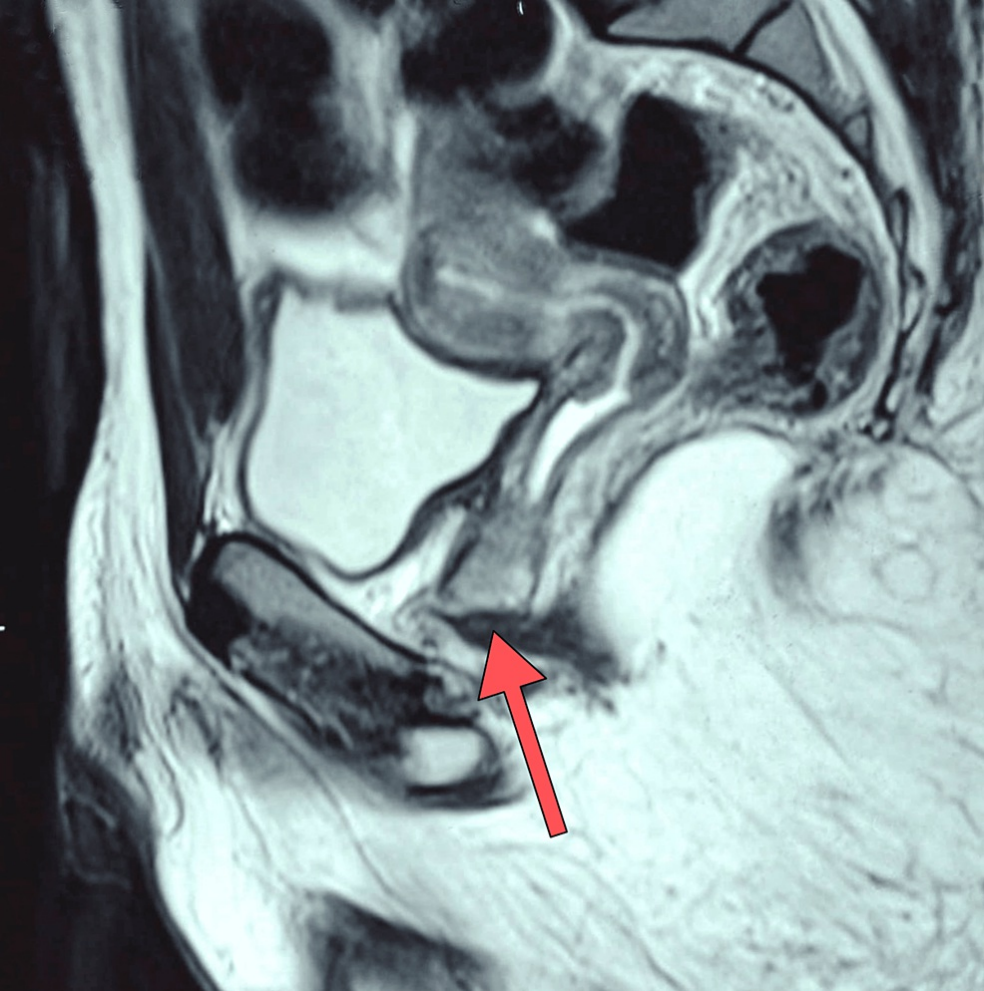
Magnetic resonance imaging of the pelvis. The uterus and vagina can be seen. Also, the abnormal connection between the vagina and posterior part of the urethra (red arrow) can be demonstrated.

MRI of the abdomen and pelvis corroborated the findings of adrenal gland enlargement and provided visual confirmation of the uterus and ovaries, which matched the measurements from ultrasonography. The MRI also confirmed the communication between the vagina and the posterior part of the urethra (Figure 4).

The typical clinical features, biochemical profile, and presence of hypertension led to the diagnosis of 46,XX CAH due to 11βOHD.

Treatment

The patient was started on oral dexamethasone (0.5 mg, hora somni) supplementation. Hypokalemia was corrected with potassium chloride syrup (10 ml, thrice daily), and hypertension was controlled using a combination of oral hypertensives, nifedipine (10 mg, twice daily) and spironolactone (25 mg, twice daily). Detailed psychiatric assessment was done and found that the patient preferred a male gender identity. The patient was admitted for gender reassignment surgery. Total abdominal hysterectomy and bilateral salpingo-oophorectomy were done. The procedure and the immediate postoperative period were uneventful. The patient was discharged one week after the surgery, with advice to return after one week for follow-up.

Follow-up

On follow-up, two weeks after the surgery, the scar at the operative site was healthy with no discharge. Sutures were removed. Blood pressure was adequately controlled, and the potassium levels had normalized. Therefore, nifedipine and potassium chloride syrup was discontinued. Sex steroid profile revealed findings as given below (Table 2). The testosterone levels were in the reference range for a female. The patient was advised to continue with oral dexamethasone (0.5 mg, hora somni) therapy and spironolactone (25 mg, twice daily). Intramuscular injection of testosterone (100 mg) once every two weeks was also initiated. Long-term telephonic follow-up showed the patient was on regular therapy and had no complaints. 

**Table 2:** Sex steroid profile results obtained during the follow-up visit.

Parameter	Value	Reference values
Serum luteinizing hormone (mIU/mL)	50.63	Male: 1.7-8.5; female: 2.4-12.6
Serum follicle-stimulating hormone (mIU/mL)	76.67	Male: 1.5-12.4; female: 3.5-12.5
Serum testosterone (ng/dL)	24.62	Male: 249-838; female: 2.9-40
Serum estradiol (pg/mL)	17.31	Male: 25-80; female: 12.4-233

## Discussion

11β-Hydroxylase occurs in the form of two isoenzymes in the adrenal cortex. These enzymes are encoded by the CYP11B1 and CYP11B2 genes, which comes under the cytochrome P450 system, on the human chromosome 8q. Among the two, the first is expressed at high levels in the normal adrenal gland, has 11β-hydroxylase activity, and is under the regulation of adrenocorticotropic hormone (ACTH). The second isoenzyme is expressed at low levels in the adrenal gland [3].

Adrenal steroidogenesis is a dynamic process that depends on de novo synthesis from cholesterol and is under the influence of ACTH and other regulatory factors. The synthesis of mineralocorticoids, glucocorticoids, and adrenal androgens occurs in distinct adrenal cortical zones, each of which expresses specific enzymes responsible for these processes. 11β-Hydroxylation is an important step in the biosynthesis of both aldosterone and cortisol. Aldosterone synthesis occurs in the zona glomerulosa, and this is the only zone that expresses CYP11B2. 11β-Hydroxylation (CYP11B2) is required to convert 11-deoxycorticosterone to corticosterone which then goes on to form aldosterone. The glucocorticoid, cortisol, is synthesized in the zona fasciculata. 11β-Hydroxylation (CYP11B1) plays a pivotal role in the conversion of 11-deoxycortisol into cortisol [4].

CAH, arising from the deficiency of 11β-hydroxylase, results from mutations in CYP11B1 encoded on chromosome 8q21. This gene includes nine exons and encodes a protein made up of 503 amino acids. Deactivation of this gene leads to the reduced activity of 11β-hydroxylase, which then leads to decreased cortisol secretion and the accumulation of glucocorticoid and mineralocorticoid precursors, and excessive adrenal androgen biosynthesis [5].

11βOHD is characterized by decreased levels of cortisol and aldosterone, accompanied by an excess of androgens. The decreased levels of cortisol trigger a feedback response to increase ACTH. Due to excess ACTH and forward blockade to the precursors in the pathway, due to 11βOHD, there is accretion of these precursors in the adrenal cortex. Ultimately, these precursors get shunted away to form adrenal sex steroids, namely, androstenedione and dehydroepiandrosterone, causing precocious puberty and virilization of female fetuses, as seen in this patient. It also contributed to the earlier development of secondary sexual features. The early onset of puberty contributed to the short stature in this patient. Though there is a decrease in the levels of aldosterone in 11βOHD, 11-deoxycorticosterone, a precursor with mild mineralocorticoid effect, gets significantly piled up in the adrenal cortex.

Mineralocorticoids work on the distal parts of the nephrons, the distal collecting duct, and the collecting tubule, by promoting sodium and water retention while also promoting potassium excretion. 11-Deoxycorticosterone, by the virtue of its weak mineralocorticoid effect, would have acted upon this patient's renal tubules to cause sodium and water retention, bringing about an expansion of the extracellular volume, leading to the development of elevated blood pressure. Furthermore, it would have also promoted the excretion of potassium in the urine paving the path for hypokalemia to set in [6].

Comparing and contrasting features of 11βOHD and 21OHD are given in the table below (Table 3). Hypertension is a feature unique to 11βOHD, like seen in this patient, through the mechanism discussed above.

**Table 3:** Comparing and contrasting features of 21OHD and 11βOHD. The differentiating factor between the two disorders is the presence of hypertension in 11βOHD. Also, there is a discrepancy between the level of potassium. Lower levels of potassium in the case of 11βOHD can be explained by the excess of 11-deoxycorticosterone, a weak mineralocorticoid. ↓: decrease; ↑: increase; +: positive

Enzyme deficiency	Aldosterone	Potassium	Blood pressure	Cortisol	Sex hormones	Post-natal virilization
21-Hydroxylase	↓	↑	↓	↓	↑	+
11β-Hydroxylase	↓	↓	↑	↓	↑	+

The cyclical hematuria and breast enlargement must have been due to menstrual cycle, as expected to occur in a normal female of reproductive age. The hematuria must have been due to the shedding of the endometrium, but instead of bleeding per vaginum, there was bleeding via the phallus due to the abnormal connection between the vagina and the posterior part of the urethra, as demonstrated in the imaging studies. The initial follow-up testosterone levels were in the reference range for a female (Table 2), as the levels of ACTH and adrenal sex steroids started falling with the supplementation of corticosteroids.

Sufficient evidence for CAH being linked with elevated serum calcium concentrations is available in the annals of medical literature. But, the mechanism of elevated levels of calcium still remains a mystery. CAH also increases the risk for hypercalciuria and nephrocalcinosis. These facts explain the medullary nephrocalcinosis in this patient [7].

The Y chromosome has the SRY gene which encodes for the testis-determining factor. This testis-determining factor is absolutely necessary for the development of testes from the immature gonads, and in the absence of the Y chromosome and the SRY gene, the immature gonads go on to develop into ovaries. Thus, this patient, due to the absence of Y chromosome, had an empty scrotum and did not even have any signs of undescended testis on examination but, instead, had a pair of ovaries as visualized in the imaging studies [8]. 

Hormone replacement has been made possible with oral glucocorticoid and oral mineralocorticoid pills, but it still can't exactly be a physiological replacement as these pills cannot accurately mimic the circadian rhythm of the adrenal cortex that is seen in a normal adrenal gland. Several new potential treatments for CAH are emerging to battle this difficulty [9].

Usually, cases of CAH are detected early. The median age of CAH diagnosis in patients is less than six months [10]. But our patient presented very late at 20 years of age, making our case report stand out from the rest.

## Conclusions

In short, this is a case of 11βOHD in the adrenal cortex which led to developmental abnormalities of the gonads in the form of the presence of a phenotypically male genitalia in a patient with an XX female genotype due to excessive virilization. It also led to precocious puberty and early-onset hypertension. Also, the patient had rudimentary ovaries, uterus, and vagina, the inferior portion of which was connected with the posterior part of the urethra. This abnormal communication is the reason for cyclical hematuria in this patient, who also has a first-degree relative with similar complaints. 

Neonatal screening programs in various countries include screening for CAH. Yet, the diagnosis of CAH is often missed in many developing countries, because of lack of awareness and financial constraints. The role of early diagnosis and treatment cannot be stressed enough for the potential well-being of those affected. Early management also helps to prevent various complications including adrenal crisis, precocious puberty, and growth failure. Our case presented very late, but with the help of gender reassignment procedures and appropriate supplementation, the patient was enabled to live a merrier life.
